# Account of the Hospitals at Berne, in Switzerland

**Published:** 1811-05

**Authors:** 


### *For the Medical and Physical Journal.*

### *Account of the Hospitals at Berne, in Switzerland.*

(Du Journal Général de Médicine, &c.)

THERE are in Berne two hospitals, one of which is appropriated to the sick, and the other to the infirm and aged. These are handsome buildings, finely situated, and appear to be well conducted.

The hospital for diseases has been built about forty years, and is formed on the plan of those in England. It contains several several airy wards of different sizes, and is capable of receiving 100 patients. The bedsteads are of wood, and without curtains.

Patients in this hospital are divided into two classes. One comprehends all those cases which are under the immediate management of the physician; the other takes the cases in surgery. Each class has its appropriate ward. There is also a ward set apart for children; a circumstance of much importance, on account of the frequency of strumous affections. Several attendants (infirmières) are attached to each ward; and a man and his wife sometimes undertake the care of the men's ward, where the most severe chirurgical cases are placed. It is common in Switzerland for men to become nurses *(garde-malades)*; and even in several towns they get their living by undertaking this employment in private families.

Two surgeons and a physician are attached to this hospital. The surgeons have the appointment for life, but the physicians, of which there are four, attend alternately each three months. They receive from the government an annual salary of 1200 francs. There has lately been added to this establishment hot and cold baths, and a small ward for unusually severe cases, and particularly for epileptics, and cases of insanity so recent as to afford hopes of cure.

The *Hôpital de la Ville* is upon a much larger scale than the preceding: it is a fine building, has a court before it planted with trees and flowers, and is divided into several quarters. Two wards are set apart for the reception of sick servants belonging to families both of the town and country: and others are appropriated to the reception of beggars, &c. who receive board and lodging for one night. The aged and infirm occupy other parts of this hospital, and there is a range of apartments, let at a small price to poor citizens capable of going out to work, but who likewise receive assistance from this public charity. The magistrates and physicians visit daily, and the whole establishment is kept in excellent order.

At half a league from the town there are three other hospitals, under the care of the same physicians, but whose interior administration belongs to the canton. These are old and inconvenient, and are inferior in every respect to the preceding establishments. They are considered as hospitals of ease to the others; one receives the insane patients, the other the syphilitic, and the third is appropriated to epileptics, cancerous, and incurable cases of scrofula, &c.

The first of these contained in August, 1805, sixty incurable lunatics, each in a separate apartment: almost the whole of these had the hair and eyes black, and were melancholy.

